# Editorial: Bridging the Gap in Neuroelectronic Interfaces

**DOI:** 10.3389/fnins.2020.00457

**Published:** 2020-06-03

**Authors:** Ulrich G. Hofmann, Jeffrey R. Capadona

**Affiliations:** ^1^Medical Center, University of Freiburg, Freiburg, Germany; ^2^Department of Biomedical Engineering, Case Western Reserve University, Cleveland, OH, United States; ^3^Advanced Platform Technology Center, Louis Stokes Cleveland Veterans Affairs Medical Center, Cleveland, OH, United States

**Keywords:** brain-machine interfaces, brain implantable devices, brain stimulation, brain recordings, chronic implants, flexible substrate, BBB rupturing, neuroprotection

In the field of Neuroelectronic Interfaces it seems as though the lines between reality and science-fiction/fantasy are often blurred. One of the inspirations for our most recent Gordon Research Conference in March 2018 aimed at “Bridging the Gap in Neuroelectronic Interfaces” dates back to 1999 when Chapin et al. (1999) described their ability to predict movement trajectories of rodents and non-human primates by “eavesdropping” on groups of neurons. Many in the field felt that science fiction seemed to become reality and the future of prosthetics appeared on the horizon. Less than a decade later invasive micro-electrode arrays and the latest jewels of micro-machining found their way into the brains of a few human patients as well (Hochberg et al., 2006), very much triggering expectations of a coming Golden Age of Brain-Machine-Interfacing for severely handicapped patients.

Unfortunately, after this very promising start two decades ago, these technologies were “lost in translation” (Ryu and Shenoy, 2009) on the way to clinical applications and widespread use. The unclear path through this first Valley of Death toward true chronic BMIs caused despair in patients and funders as well. It became clear a deterioration of signal quality largely due to the brain's fierce response to foreign bodies leads to a loss in high quality, wide-band signals meant to control any artifact or prosthetic device. One major obstacle thought to limit practical clinical translation is the poor understanding of failure modes of all types of high channel count implanted microelectrode arrays and how to counteract them. Besides fabrication and handling related failures (abiotic), several classes of multi-modal problems were encountered (biotic). The strong sterile inflammation and thus an electrical decoupling of implanted devices from the brain were identified as the major obstacle on the path to chronic applications in humans. However, where were the ideas to overcome this hurdle?

Such was the frustrating situation in our field when we were honored in 2016 by the Gordon Research Organisation to organize and run their inaugural conference dedicated to the unexpectedly complex neuro-electronic interface. Our intention was to look beyond the usual trinity of neurons, astrocytes, and microglia and provide some type of common knowledge in the surrounding of our challenge. The conference took place in 2018 in Galveston, Texas, and was attended by almost 200 researchers. It had an exquisite line-up of excellent speakers, motivated discussion leaders and curious audience—in short, it was a full week of intense discussion, collective brainstorming and shared experience—it was truly a success!

However, rules of the Gordon Research Conferences series prohibits documentation in any form, no photos, no abstracts were published—only paper and pencil are welcome. Therefore, this Special Research Topic is supposed to partially remedy this lack of collected knowledge and provide the multi-disciplinary audience of leading experts in micro-technology, cellular neuroscience, brain pathology, neuro-engineering, and materials science a platform to present their cutting edge solutions after peer review. We surely believe that this collection will progress the quest for a chronically useful and reliable neural interface.

Several insightful reviews support our conference's look outside the box and give an overview into microfluidic based model systems (Gulino et al.) useful to further explore the foreign body responses or nano-particle based stimulations. The prospects of bioactive, so called “living electrodes” were discussed by Adewole et al., who points out several differing approaches to “trick” the brain into incorporating artificial implants. The biomechanics of neuronal adhesion may play an important role in this context and can be assessed by frequency analysis of quartz crystal oscillators, as is pointed out by Khraiche et al.. As important as microscopic biomechanics is in our field of research, it is equally difficult to quantify in brain tissue. Help is offered from localized probing of biomechanics by Atomic Force Microscopy (AFM) as is briefly reviewed by Viji Babu and Radmacher. Another exciting review by Aplin and Fridman extensively discusses the rarely used constant current (DC) stimulation of neural tissue, a potential new field of neuromodulation enabled by recent developments in microfluidics.

As one common denominator, several groups reported on recent approaches to a long-standing idea to use of flexible or compliant substrates. For example, Hosseini et al. went so far as to present a novel, more stable, shape-memory polymer, able to soften in an aqueous environment. Materials of this type are designed to remain stiff during implantation, but compliant when deployed in the brain. Implantation of a stiff microelectrode may suffice to express c-Fos, a popular early marker for neuron activation, as found by Pflüger et al.. Flexible substrates were also presented by Dorand et al. and Reddy et al., exhibiting double sided electrodes to create novel micro-LEDs toward optogenetic applications. Optogenetic applications were the focus of another study that reported on an important milestone toward clinical use (Williams et al.). They were able to show optogenetic stimulation of muscles in non-human primates! It goes without saying, flexibility was not a feature expected from silicon probes successfully used in large animals as reported by Ulyanova et al..

Electrode composition and performance was an issue investigated in several articles.

Meijs et al. deposited different layers of Boron Doped Diamond (BDD) on TiN electrodes and compared them electrochemically, identifying good candidates for further *in vivo* testing. The article from Ferlauto et al. demonstrates a reduction of electrical noise by inserting conductive polymers as compliant intermediary on Pt-electrodes. Whereas, the work by Neto et al. informed us to stop worrying about impedances—at least of recording micro-electrodes in the usual range (0.1 to 2 MΩ)—as long as they exhibit a low shunting capacity. In contrast, potentiostatic experiments done by Harris et al. concluded that the use of Ohm's law to describe electrical stimulation over Pt-electrodes is an unwarranted oversimplification, ignoring the electrically complex, spatially varying tissue-electrode interface. In order to further the quality control with MEAs Suarez-Perez et al. introduced spectral definitions of SNR based on cortical slow oscillations (SO) providing a less disputable “signal” (LFP UP state) over a “noise” state.

Several articles dealt with improvements for artificial sensing front ends:

Losada et al. took the well-known mushroom electrodes (Spira and Hai, 2013) to a new level, by placing them on a flexible substrate inside a cavity, improving the stimulation efficacy to bipolar retinal cells. A more traditional approach to the retina was taken by Rincon Montes et al. who used custom designed, laminar and penetrating silicon probes to assess stimulation effects on other retinal layers in an attempt to close the stimulation loop. Ryu et al. went in a similar direction, but separated retinal electrical stimulation from laminar visual cortex recordings. Stimulation of retinal ganglion cells was investigated both by simulation and *in vitro* experiments to shed light on their non-monotonic response profile to high-frequency stimuli (Guo et al.). A novel simulation tool was presented by Al Abed et al. to shed light on *in vivo* electroporation in context of gene therapeutic improvements of the cochlea-electrode-interface.

Improvements of optical techniques were presented by several other articles.

Nambiar et al. demonstrated an algorithmic pipeline to reconstruct brain tissue surrounding explanted “hybrid” array electrodes. Esquibel et al. employed the label free, optical sectioning method of second harmonic generation to examine implanted brain slices and showed unusual collagen fiber patterns not found in normal brains. Quite remarkably, by applying a custom made optoacoustic imaging setup Gottschalk et al. monitored neuronal calcium dynamics under blood-free conditions deep in an *ex vivo* maintained whole mouse brain. It will not be the last we are going to hear from genetically encoded calcium indicators (GECI). Improvements in 2-photon imaging presented in the review by Dorand et al. show a complicated and dynamic response to BBB-rupturing, substantial immune activation and microglia participation which might warrant a systematic application of different medications. Wellman et al. further supports the quest to widen the circle of usual suspects around brain implanted devices as they reveal an involvement of a wealth of other players like oligodendrocytes or even pericytes. A view shared by Bedell et al. and Hermann et al. poihc who not only vote for minimizing the cross sectional area of implants, but propose benefits from targeting the TLR/CD14 pathway as a therapeutic mechanism—in particular focusing on infiltrating peripheral immune cells, while allowing the resident microglia to facilitate neuroprotection.

As hoped for while organizing the conference, several overarching cutting-edge topics were presented to the community for the first time. The conference organizers then crystalized the momentum from the meeting into in the subsequent articles reported in this virtual issue. Unfortunately, the COVID-19 pandemic postponed the most recent installment of our meeting. Please continue to check the Gordon Research Conference website for information about the rescheduled conference.

## Author Contributions

UH and JC both wrote and edited this article.

## Conflict of Interest

The authors declare that the research was conducted in the absence of any commercial or financial relationships that could be construed as a potential conflict of interest.
